# The Prognostic Value of Toll-Like Receptors in Head and Neck Squamous Cell Carcinoma: A Systematic Review and Meta-Analysis

**DOI:** 10.3390/ijms21197255

**Published:** 2020-09-30

**Authors:** Shrabon Hasnat, Roosa Hujanen, Bright I. Nwaru, Tuula Salo, Abdelhakim Salem

**Affiliations:** 1 Department of Oral and Maxillofacial Diseases, Clinicum, University of Helsinki, 00014 Helsinki, Finland; shrabon.hasnat@helsinki.fi (S.H.); roosa.hujanen@helsinki.fi (R.H.); tuula.salo@helsinki.fi (T.S.); 2 Krefting Research Centre, Institute of Medicine, University of Gothenburg, 40530 Gothenburg, Sweden; bright.nwaru@gu.se; 3 Wallenberg Centre for Molecular and Translational Medicine, Institute of Medicine, University of Gothenburg, 40530 Gothenburg, Sweden; 4 Translational Immunology Research Program (TRIMM), Research Program Unit (RPU), University of Helsinki, 00014 Helsinki, Finland; 5 Cancer and Translational Medicine Research Unit, University of Oulu, 90220 Oulu, Finland; 6 Medical Research Centre, Oulu University Hospital, 90220 Oulu, Finland; 7 Helsinki University Hospital (HUS), 00029 Helsinki, Finland

**Keywords:** toll-like receptors, head and neck squamous cell carcinoma, cancers, metastasis, meta-analysis, biomarker, prognosis

## Abstract

Head and neck squamous cell carcinoma (HNSCC) is a group of tumours which exhibit low 5 year survival rates. Thus, there is an urgent need to identify biomarkers that may improve the clinical utility of patients with HNSCC. Emerging studies support a role of toll-like receptors (TLRs) in carcinogenesis. Therefore, this systematic review and meta-analysis was performed to assess the prognostic value of TLR immunoexpression in HNSCC patients. We compiled the results of thirteen studies comprising 1825 patients, of which six studies were deemed qualified for quantitative synthesis. The higher immunoexpression of TLR-1 to 5 and 9 was associated with a worsening of the clinical parameters of patients with HNSCC. Furthermore, induced levels of TLR-3, 4, 5, 7 and 9 were found to predict the patients’ survival time. The meta-analysis revealed that TLR-7 overexpression is associated with a decreased mortality risk in HNSCC patients (HR 0.51; 95%CI 0.13–0.89; I2 34.6%), while a higher expression of TLR-5 predicted shorter, but non-significant, survival outcome. In conclusion, this review suggests that TLRs may represent some prognostic value for patients with HNSCC. However, due to small sample sizes and other inherent methodological limitations, more well designed studies across different populations are still needed before TLRs can be recommended as a reliable clinical risk-stratification tool.

## 1. Introduction

Head and neck squamous cell carcinoma (HNSCC) is a group of related tumours that develop in the epithelial lining of the oral cavity, oropharynx, hypopharynx, and larynx [1]. Oral squamous cell carcinoma (OSCC), for instance, is the 12th deadliest cancer type in the world, accounting for more than 90% of all HNSCC cases [2,3]. HNSCC is mainly associated with tobacco and alcohol consumption and has recently been associated with human papillomavirus (HPV) infection [3,4]. Despite the notable advances in the treatment approaches of cancer patients, the 5 year survival rate of HNSCC patients has not significantly improved in recent decades [4]. In fact, the use of prognostic factors has been one of the important management strategies to guide the selection of an appropriate treatment plan for HNSCC patients. However, the enormous heterogeneity of HNSCC tumours has limited the reliability of most of the currently available prognostic markers [4]. Thus, there is an urgent need to identify new prognosticators in HNSCC, and to assess whether they can serve as a guide for risk stratification and treatment decisions.

Toll-like receptors (TLRs) are a group of transmembrane signalling proteins and pattern recognition receptors that are expressed by cells and are an integral part of the innate immune system [5]. Thirteen different TLRs were reported in mammals, of which 10 are expressed in humans [6]. Each of the TLRs are characterised with distinctive functions in the body, e.g., TLR-2 and TLR-4 identify lipopolysaccharide membrane components of Gram-negative bacteria, TLR-5 recognises bacterial flagellin, TLR-7 identifies microbial nucleic-acid structures, while TLR-9 identifies microbial DNA-strands [5]. In this regard, TLR-activating ligands are divided into two categories: pathogen-associated molecular patterns (PAMPs), which are found in exogenous pathogens such as in microbes (viruses, bacteria, etc.); and damage-associated molecular patterns (DAMPs), which are endogenously expressed from injured or dying cells to regulate apoptosis, thereby upkeeping homeostasis [7].

Clinical studies showed that functional TLRs are expressed in a wide variety of malignant tissues suggesting a crucial role of TLRs in tumorigenesis [8]. However, TLR signalling, by tumour and immune cells, was shown to have a dual effect—inducing both tumorigenic and antitumorigenic effects [7,8]. For instance, it has been suggested that TLRs expressed on immune cells, during inflammation, can mediate antitumoral effects, while these expressed on tumour cells can enhance cell proliferation and anti-apoptotic effects during tumorigenesis [7].

Despite the intensive efforts to examine the usefulness of TLR expression as a survival prognosticator in cancer patients, the results of many prognostic studies are not always consistent. In this regard, meta-analysis compiles previous studies, and enhances the statistical power by pooling the data from independent analyses [9]. Thus, in this study, we conducted a systematic review to identify and critically appraise studies that have investigated the association between TLR immunoexpression and the survival of HNSCC patients to date. In addition, we performed quantitative synthesis to assess whether TLR immunoexpression can be used as a useful prognostic marker in HNSCC.

## 2. Results

### 2.1. Study Selection

**Figure 1:**
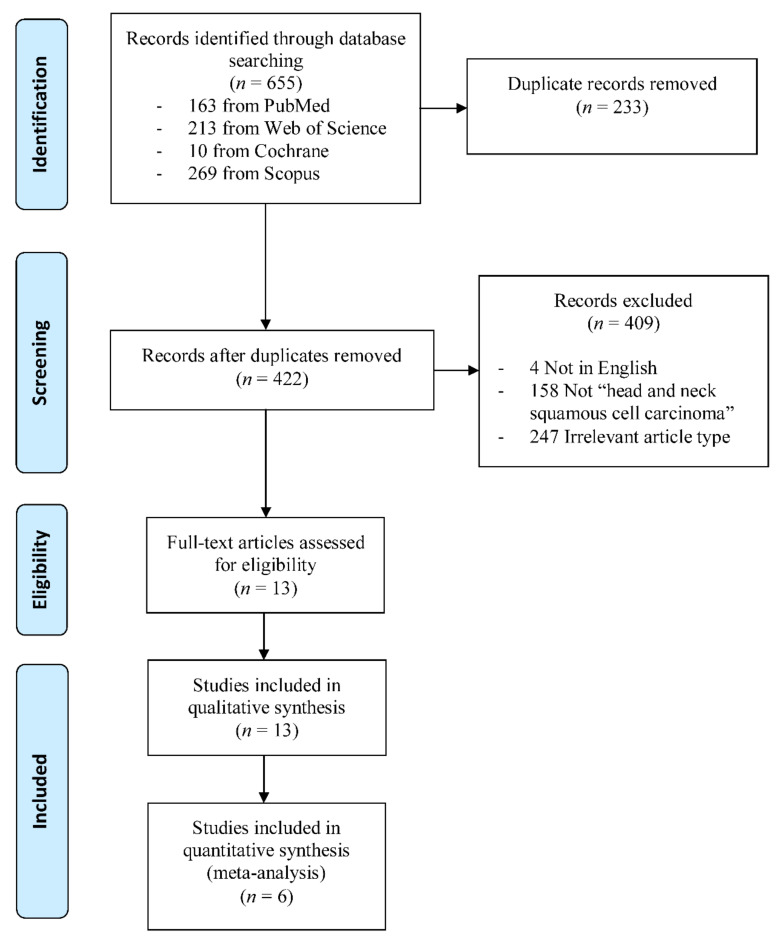
Flowchart diagram of the literature search and selection process.

The database search yielded a total of 655 studies. After the removal of duplicates, the titles and abstracts of the remaining studies were screened against the eligibility criteria and 409 studies were excluded as irrelevant. Then, the thirteen remaining studies were selected for further qualitative analysis. Of these, six studies were deemed eligible to be analysed by the statistical synthesis. The literature searching and screening process is illustrated in Figure 1.

### 2.2. Study Characteristics

Thirteen original research studies encompassing a total of 1825 patients were included in this study. These studies were published between 2013 and 2020, and they were conducted in Finland (*n* = 6), China (*n* = 5), Sweden (*n* = 1), and Germany (*n* = 1). The HNSCC histological subtypes included oropharyngeal squamous cell carcinoma (OPSCC) [10,11], oral squamous cell carcinoma (OSCC) [12,13,14,15,16], nasopharyngeal carcinoma (NPC) [17], base-of-tongue squamous cell carcinoma (BOTSCC) [18], and oral tongue squamous cell carcinoma (OTSCC) [19,20,21]. Long et al. studied samples from tongue SCC (TSCC), however, it was not specified whether they were obtained from OTSCC or BOTSCC tissues [22]. The sample size per study ranged between 60 and 207 patients. The immunostaining in these studies was conducted on formalin-fixed paraffin-embedded samples, and targeted the following TLR subtypes: TLR-1 [17,22], TLR-2 [17,20], TLR-3 [12], TLR-4 [13,15,17,20], TLR-5 [10,11,16,17,18,20,21], TLR-7 [11,14,17,18,20], and TLR-9 [11,17,19,20]. The main characteristics of the included studies and the staining reagents are depicted in Table 1 and Table 2, respectively.

### 2.3. Quality and Bias Assessment

For the quality assessment, five studies (38%) fulfilled all of the applied Reporting Recommendations for Tumor Marker Prognostic Studies (REMARK) criteria [10,11,17,19,21], while the eight remaining studies had one or more missing items of the applied REMARK checklist (Supplementary Table S1). Results from the Meta-Analysis of Statistics Assessment and Review Instrument (MAStARI) critical appraisal tool revealed that all the included studies had no considerable reporting bias. The risk of bias was moderate in two studies (15%) [12,21], and low in the remaining eleven studies. Additional information can be found in Supplementary Table S2.

### 2.4. The Cutoff Values

The included studies employed different scoring methods to determine the cutoff values, including: 1) immunoreactive scoring (IRS), with grading from 0 to 12 [13], and IRS grading from 1 to 4 [22]; 2) a histoscore was calculated by multiplying the intensity score by the percentage of positive cells, resulting in a number between 0 and 300 [19,21]; 3) the receiver operating characteristics (ROC) analysis was carried out by one study based on the highest diagnostic average of sensitivity and highest diagnostic accuracy [16]. More information regarding the cutoff values and staining methods are listed in Table 2.

### 2.5. TLR and Clinicopathological Parameters of HNSCC Patients

The expression of TLR-1 was significantly correlated with advanced tumour–node–metastasis (TNM) staging (*p* < 0.05) in patients with TSCC [22]. In NPC, TLR-1 positivity was strong in 82% of the Finnish patient cohort, however, it had no correlation with the patients’ characteristics [17]. The expression of TLR-2 was also induced in 133/141 (94%) of the same cohort of NPC, and it was significantly correlated with older patient age (*p* = 0.036) [17]. In another Finnish cohort of 73 OTSCC patients, the higher expression of TLR-2 was correlated with deeper tumour invasion and grade (*p* = 0.026; *p* = 0.021, respectively) [20]. A recent study by Han et al. reported that the higher expression of TLR-3 was correlated with poorly differentiated tumour grades in OSCC patients [12]. In another cohort of Chinese OSCC patients, cytoplasmic TLR-4 expression was significantly increased in the tumour tissues and was correlated with deeper tumour invasion (*p* = 0.008), poor differentiation (*p* = 0.034), and an advanced pathologic TNM stage (*p* = 0.008). [13]. In accordance with these findings, Mäkinen et al. found that a higher TLR-4 expression in OTSCC tissues was correlated with deeper tumour invasion (*p* = 0.008) and higher tumour grade (*p* = 0.005) [20]. Likewise, another study by Ren et al. reported a significant correlation between the higher TLR-4 expression with tumour T-stage (*p* = 0.005), clinical stage (*p* = 0.005), histological classification (*p* = 0.023) and lymph node metastasis (*p* = 0.014) [15].

Interestingly, TLR-5 was the most studied subtype in the included reports. Recently, Kylmä et al. found that higher TLR-5 immunoexpression was associated with advanced N-class (N1–N3; *p* = 0.008), tumour site (tonsil; *p* = 0.006) and advanced tumour grade (grade 3; *p* = 0.004) in OPSCC tissues [10]. In another Finnish cohort of OPSCC patients, TLR 5 expression was more induced among current smokers (*p* < 0.001) and alcohol abusers (*p* = 0.002), but less expressed in T1 tumours and regionally advanced metastatic tumours of OPSCC patients [11]. However, although TLR-5 was associated with older NPC patients, no statistically significant associations were found between TLR-5 and smoking, TNM classification, or overall stage [17]. In OTSCC, a higher TLR-5 expression correlated with lower tumour grade (*p* = 0.039) [20]. Grimm et al. found that the TLR-5 expression was not associated with any clinicopathological characteristics of OSCC patients [16]. In OTSCC, higher TLR-5 was associated with older age (>70 years at the time of diagnosis), female gender and disease recurrence (*p* < 0.05) [21]. On the contrary, no association between TLR-5 expression and tumour grade, stage or treatment was found in OTSCC [21].

The expression of TLR-7 was significantly low in OPSCC patients with a history of smoking and alcohol abuse [11]. However, higher TLR-7 was correlated with tumour site (*p* = 0.004), regional metastasis (*p* = 0.001) and advanced stages (*p* = 0.003) in patients with OPSCC [11]. In the same study, higher TLR-9 expression was associated with current smoking (*p* < 0.001), but not with any other clinical parameters [11]. Kauppila et al. found that higher TLR-9 immunoreactivity in OTSCC was associated with poor tumour differentiation (*p* < 0.05) [19].

### 2.6. TLR and the Viral Status of HNSCC Patients

Five studies assessed the association between TLRs and the viral status of human papilloma virus (HPV) and/or the Epstein–Barr virus (EBV) in HNSCC patients [10,11,17,18,20]. Kylmä et al. found that higher TLR-5 expression was associated with poor disease-specific survival (DSS) in HPV-positive OPSCC patients [10]. In agreement with this, higher TLR-5 and lower TLR-7 expression had worse recurrence-free survival (RFS) and DSS in another Finnish cohort of OPSCC patients [11]. In NPC patients, it was found that only TLR-2 and TLR-5 expressions were related to viral status, where their expression was stronger in the HPV-positive and in the EBV/HPV-negative patients than in the EBV-positive group (*p* < 0.0001) [17]. On the contrary, TLR-5 expression was markedly weaker in HPV-positive BOTSCC patients (*p* < 0.001), while the opposite was observed for TLR-7 (*p* < 0.007) [18]. Mäkinen et al. reported that p16^INK4a^, a surrogate marker for HPV, did not correlate with TLR expression in OTSCC patients [20].

### 2.7. TLR and the Survival Outcomes of HNSCC Patients

Five studies analysed the overall survival (OS) as the primary survival endpoint [12,13,14,17,20]. Other endpoint measures included disease-free survival (DFS), DSS (or cancer-specific survival, CSS) and/or RFS. Study-specific endpoints are listed in Table 3. One report by Han et al. indicated that higher TLR-3 expression was associated with shorter OS [12]. Likewise, higher TLR-4 expression predicted shorter OS, DFS, DSS and post-operative survival in two Chinese studies conducted on patients with OSCC [13,15]. A strong expression of TLR-5 predicted worse DSS and RFS in HPV-positive in Finnish OPSCC patients [10,11]. Furthermore, a higher TLR-5 expression in OTSCC patients was associated with reduced DFS outcomes [21]. However, Grimm et al. found no association between TLR-5 expression and DFS in OSCC patients [16].

For TLR-7, Ruuskanen et al. found that NPC patients with a positive expression had better OS than those with negative TLR-7 [17]. Interestingly, according to TLR-7 expression, the 5 year OS rates were 66% (for mild expression) and 22% (for negative status) [17]. Moreover, the low expression of TLR-7 predicted poor DSS and RFS in HPV-positive OPSCC patients [11]. Ni et al. reported that the higher expression of TLR-7 in tumour cells was correlated with shorter OS in OSCC [14]. On the contrary, higher TLR-7 in the stromal fibroblast-like cells (FLCs) correlated with a longer survival time than the low expression group [14]. However, no statistically significant correlation was found between TLR-5 and 7 with the clinical outcome or survival in BOTSCC patients [18]. One study revealed that strong TLR-9 expression was an independent predictor of poor CSS [19].

### 2.8. Meta-Analysis Results

**Figure 2:**
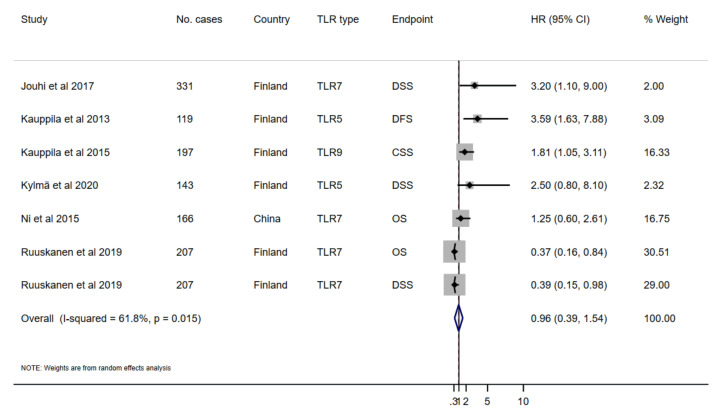
Forest plot of the association between toll-like receptors (TLR) and survival outcomes in HNSCC patients (random-effects model). HR, hazard ratio; CI, confidence intervals.

**Figure 3:**
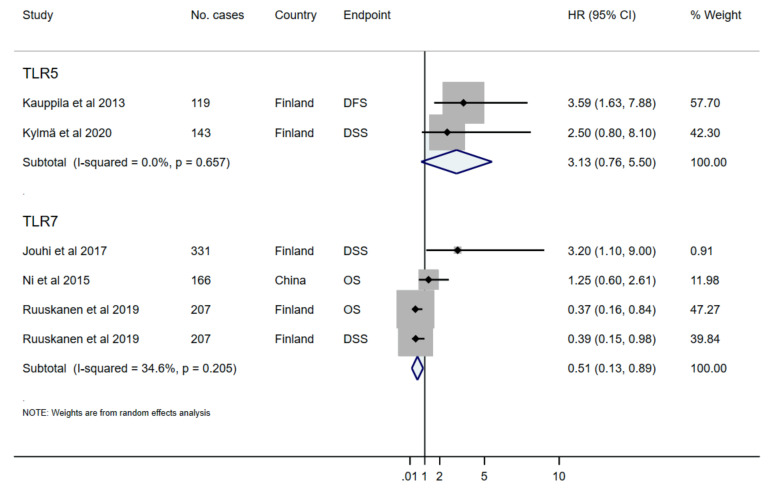
Forest plot demonstrating a subgroup analysis of the association between the studied subtype of toll-like receptor (TLR) and survival outcomes in HNSCC patients (random effects model). HR, hazard ratio; CI, confidence intervals.

Among thirteen studies selected for the qualitative analysis, seven studies were excluded because of insufficient data for quantitative synthesis. When the remaining six studies were combined, regardless of the TLR subtype and endpoint, TLR was associated with a small non-statistically significant reduced survival (HR 0.96, 95% CI 0.39–1.54; I^2^ 61.8%, *p*-value for I^2^ = 0.0015) (Figure 2; Table 4). Then, we performed a subgroup analysis of the studies based on the reported TLR subtype. Studies reporting TLR-5 (*n* = 2) and TLR-7 (*n* = 4) were adequately powered and eligible for the pooled analysis. As a result, higher TLR-5 expression was associated with an increased non-statistically significant risk of mortality (HR 3.13, 95%CI 0.76–5.50; I^2^ 0.0%, *p*-value for I^2^ = 0.657), while higher TLR-7 was associated with a statistically significant decreased risk of mortality in HNSCC patients (HR 0.51, 95%CI 0.13-0.89; I^2^ 34.6%, *p*-value for I^2^ = 0.205) (Figure 3; Table 4). Studies were further analysed according to the survival endpoints, however, it was not possible to obtain meaningful results because of the differences in the applied scoring categories per respective measure. 

## 3. Discussion

**Figure 4:**
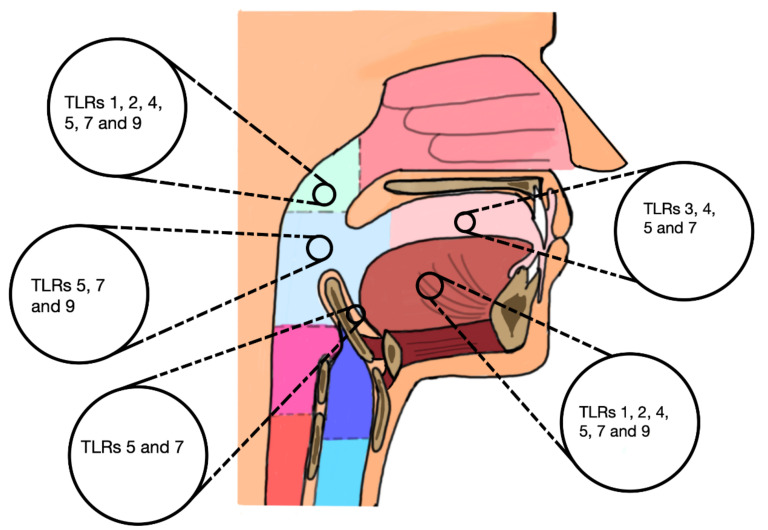
An illustration of the studied toll-like receptors (TLRs) in the corresponding tumour site of the head and neck squamous cell carcinoma patients included in the present review.

In the present meta-analysis, we compiled and summarised the results of thirteen clinical studies comprising a total of 1825 patients, of which six studies with collectively 1163 patients were deemed qualified for further quantitative synthesis. These studies assessed the prognostic value of TLR-immunoexpression in HNSCC (Figure 4). A higher immunoexpression of TLR-1 to 5, 7, and 9 was significantly correlated with at least one worsening clinical parameter and/or the viral status of patients with HNSCC. Furthermore, a shorter survival outcome was predicted by an induced expression of TLR-3, 4, 5 and 9, while TLR-7 predicted a favourable prognosis in most relevant studies. This was further analysed by subgroup meta-analysis, which confirmed the prognosticator role of TLR-7 in HNSCC. The higher expression of TLR-5 predicted shorter survival outcomes, however, our meta-analysis revealed no significant evidence to support such a role. Likewise, there were no statistically significant effects of TLRs when all studies were combined, regardless of the receptor subtype.

To our knowledge, this is the first meta-analysis report assessing the prognostic value of different TLRs in patients with HNSCC. Among the strengths of our study that our review protocol was developed as recommended by PRISMA guidelines, which was registered in PROSPERO prior to conducting this study. In addition, we were able to conduct quantitative analyses due to an adequate number of eligible studies. Most of these studies fulfilled, by and large, the applied REMARK criteria and exhibited low scores of reporting bias (Table 2 and Supplementary Table S2). Furthermore, there was less variation among studies regarding the clinicopathological features, immunodetection reagents and staining methods. However, several limitations should be considered while interpreting the results of this study, such as the small sample sizes and limited number of studies per some receptor subtypes. In addition, we were not able to find any prognostic data on TLR-6 or 8 in HNSCC patients. Due to the lack of adequate estimates, we only managed to include 46.1% of the primary studies in the quantitative synthesis. A considerable heterogeneity (I^2^ > 60%) was detected when all studies were combined, which could arise from variabilities in TLR subtype, tumour site, and the estimated endpoints. Finally, since most studies were conducted in limited populations (i.e., Finnish or Chinese), this may hinder the robustness of such association estimates. Hence, the results should be interpreted cautiously as they may bear limited feasibility for clinical stratification approaches.

TLRs are crucial mediators of inflammatory responses, which can promote carcinogenesis through different molecular mechanisms [23]. In this regard, inflammation-associated molecular patterns (i.e., PAMPs and DAMPs), which signal via TLRs, play different roles in the development of HNSCC [24]. For instance, TLR-5 recognises bacterial flagellin, which can influence tumour growth and progression in vitro [25,26]. On the other hand, the activation of TLR-7 can induce Type 1 interferon and inflammatory response, and hence represents a promising target for antiviral and antitumour therapy [27]. Indeed, tumorigenesis and tumour metastasis are complex multistep processes, which involve the differential upregulation and downregulation of multiple genetic and epigenetic signalling pathways [28]. In the present review, the clinical studies revealed differential expressions of the TLR family in various HNSCC tumours, of which TLR-3, 4, 5, 7 and 9 had certain prognostic values in their respective tumours. Recently, we also reported an altered expression of TLRs in oral lichen planus—a potentially premalignant lesion of HNSCC [29,30].

The correlation between the expression of TLR subtypes and the outcome of patients has also been investigated in other common carcinomas. Oesophageal SCC (ESCC), for instance, ranks seventh in terms of cancer incidence and shares broad similarities with HNSCC [31,32]. In ESCC, TLR-3 overexpression was significantly correlated with worse clinical parameters, while higher levels of TLR-9 were positively associated with advanced tumour grade, lymph node and distant metastases [33,34]. A similar prediction of worse outcome was also reported for TLR-4 in patients with pancreatic adenocarcinoma, where its higher expression was significantly associated with a shorter OS [35]. Interestingly, these reports are in agreement with the findings of the HNSCC studies included in this review [12,13,15,19]. However, the prognostic values of TLRs were not always consistent between different studies. For instance, Sato et al. found that ESCC patients expressing higher levels of TLR-3 had better survival outcomes [36]. Likewise, higher TLR-4 expression in ESCC patients was associated with better survival compared with weaker expression [37].

Conflicting results in the prognostication value of TLRs may likely arise from several key factors, such as differences in clinical characteristics, cancer type, cutoff values and the detection methods of TLRs. In this regard, most of the studies reported a worse survival outcome in association with a higher TLR-5 expression [10,11,21]. However, one study found that negative or mild TLR-5 expression predicted poor survival in OTSCC, while two other studies did not find any significant correlation between TLR-5 expression and patient survival [16,18,20]. These conflicting results could also be explained by the HPV status, which is probably negative in OSCC rather than OPSCC patients [11]. On the other hand, TLR-7 was associated with favourable prognosis in HNSCC, which was further confirmed by our meta-analysis. Although high TLR-7 expression in OSCC cells predicted poor survival time, Ni et al. found that high TLR-7 in stromal FLCs plays an antineoplastic role during oral carcinogenesis [14]. In fact, such a finding could in part be attributed to the promising antitumour effect of TLR-7 in various cancers, such as OSCC, breast cancer and lymphoma [38,39,40].

In conclusion, most studies in this review indicated a differential expression of TLRs in HNSCC, which was correlated with worsening clinical parameters and/or survival. Of these receptors, TLR-7 may represent a prognostic value in HNSCC, particularly for patients with OPSCC. Altogether, these findings support the putative role of TLRs in tumorigenesis. However, due to the aforementioned inherent methodological limitations, more well designed studies with larger sample sizes across different populations are needed before TLRs can reliably be recommended for clinical staging approaches.

## 4. Materials and Methods

### 4.1. Protocol and Registration

The protocol of this study was developed in accordance with the guidelines of the Preferred Reporting Items for Systematic Reviews and Meta-analyses (PRISMA) [41], which was prospectively registered in the International Prospective Register Of Systematic Reviews PROSPERO (https://www.crd.york.ac.uk/prospero/) [42].

### 4.2. Inclusion and Exclusion Criteria

Studies were eligible for inclusion when the following criteria were met: (1) original research articles conducted on human tissue samples; (2) patients diagnosed with HNSCC; (3) the relationship between TLR immunoexpression and the clinical or survival outcomes was assessed. Studies were excluded if they were case reports, case series, editorials, reviews, or involving animals. The detailed inclusion and exclusion criteria are listed in Table 5.

### 4.3. Search Strategy and Study Screening

Studies were searched from the inception until the 11th of May 2020 using the following electronic databases: PubMed, Cochrane, Scopus and Web of Science. We implemented the following search terms, adapting accordingly to the respective databases: (“Toll-like receptors” OR “Toll like receptors” OR “TLR”) AND (“head and neck neoplasms” OR “head and neck cancer” OR “head and neck squamous cell cancer” OR “oral cancer” OR “mouth neoplasms” OR “laryngeal neoplasms” OR “gingival neoplasms” OR “lip neoplasms” OR “palatal neoplasms” OR “tongue neoplasms” OR “pharyngeal neoplasms” OR “squamous”) AND (prognos* OR predict* OR surviv* OR recur* OR mortal* OR metasta*). No search restrictions were applied for the date of publication or language. The resulted articles were then imported to RefWorks, where duplicates were removed. Then, studies were screened by title and abstract to fulfil the eligibility criteria listed in Table 1. Thenceforth, full articles were retrieved for further qualitative review. The literature screening was conducted independently by two reviewers (SH and RH). A third reviewer (AS) intervened if there were any disparities between the results.

### 4.4. Data Extraction and Study Items

We first developed a data extraction form, which was used by the same two reviewers (SH and RH) to extract the following key items from the included studies: study’s title, first author name, and year of publication; patient characteristics; type of patient’s sample; tumour-related characteristics (type, stage, grade, location); the studied TLR subtype; TLR scoring/expression findings; cutoff value; antibody-related information (dilution, company, etc.); study period; follow-up durations; outcome measures; endpoint of survival analysis; and estimates of prognosis such as hazard ratio (HR) with their respective 95% confidence interval (CI) and p values, and the study’s main conclusions.

### 4.5. Assessment of Study Quality and Reporting Bias

Data reporting quality has been assessed using the Reporting Recommendations for Tumor Marker Prognostic Studies (REMARK) guidelines [43]. Six assessment items were adapted from the REMARK checklist as they were evinced to be applicable for our study, as follows: (1) patient samples; (2) clinical data of the studied cohort; (3) immunohistochemical methods; (4) prognostic and survival data; (5) applied statistics; and (6) classical prognostic factors. These applied REMARK items are detailed in Supplementary Table S1. Two reviewers (SH and RH) independently analysed the risk of bias of the included studies by employing the Meta-Analysis of Statistics Assessment and Review Instrument (MAStARI) tool as we recently described [44]. The analysis report was derived from a ten-question critical appraisal form aimed to determine the extent of bias in the study’s design, conduct and analysis (Supplementary Table S2). Discrepancies within the results were resolved by discussion with a third reviewer (AS). Discrepancies resulted in general from data overlooking or misunderstanding, and thus they were easily resolved without too much discussion.

### 4.6. Data Synthesis and Statistical Analysis

Descriptive tables were used to summarise the characteristics of the included studies, as well as presenting their derived quality grades obtained on the basis of the REMARK framework [43]. Both narrative and quantitative syntheses were performed to summarise our results. The quantitative synthesis involved conducting a random-effects meta-analysis of the studies judged to be reasonably homogenous with regards to their consistency in methodology and definitions. The random-effects model was undertaken using the DerSimonian–Laird estimate of the variance of the effect sizes. The weights assigned to the studies in the meta-analysis were based on the inverse variance method, that is, weight = 1 divided by the square of the standard error of the effect estimate. We first performed a meta-analysis of all studies combined together regardless of their TLR subtypes. Then, we performed subgroup analysis by TLR subtype (separately for TLR-5 and TLR-7 studies) and by survival endpoint. We quantified heterogeneity between studies using the I-squared (I^2^) statistic, which quantifies the percentage of variance in the pooled estimates that is attributable to differences in estimates between the meta-analysed studies rather than due to chance. We estimated the between-study variance using the Tau-squared (T^2^) statistic, which was derived from the DerSimonian–Laird estimate. All tests were 2-sided, and *p* < 0.05 was considered statistically significant. The meta-analyses were performed using Stata 14 (StataCorp. 2015. Stata Statistical Software: Release 14. College Station, TX, USA: StataCorp LP).

## Figures and Tables

**Table 1:** General characteristics of the included studies.

Study	Origin	Tumour Type	Tumour Stage	Cases	Age	Study Period	TLR	Compliance to REMARK
[10]	Finland	OPSCC	I–IV	143	HPV+ 60.8 HPV− 62.2 (M)	2012–2016	TLR-5	Fulfilled all items
[12]	China	OSCC	T1–T4	90	62.5 (M)	2005–2008	TLR-3	Lacked item No. 5
[17]	Finland	NPC	I–IV	207	57 (M)	1990–2009	TLR-1, TLR-2, TLR-4, TLR-5, TLR-7, TLR-9	Fulfilled all items
[18]	Sweden	BOTSCC	I–IV	77	62 (M)	2000–2011	TLR-5, TLR-7	Lacked item No. 5
[11]	Finland	OPSCC	I–IV	331	-	2000–2009	TLR-5, TLR-7, TLR-9	Fulfilled all items
[13]	China	OSCC	I–IV	110	60 (Med)	2006–2010	TLR-4	Lacked item No. 5
[22]	China	TSCC	I–IV	60	57 (M)	2013–2015	TLR-1	Lacked items No. 1, 4, 5
[14]	China	OSCC	I–IV	166	-	2000–2011	TLR-7	Lacked item No. 3
[19]	Finland	OTSCC	1–4	197	65 (Med)	1981–2009	TLR-9	Fulfilled all items
[20]	Finland	OTSCC	I–IV	73	59 (Med)	1992–2002	TLR-2, TLR-4, TLR-5, TLR-7, TLR-9	Lacked item No. 5
[15]	China	OSCC	T1–T4	61	59.31 (M)	1992–2007	TLR-4	Lacked items No. 4,5
[16]	Germany	OSCC	I–IV	191	-	-	TLR-5	Lacked item No. 1
[21]	Finland	OTSCC	1–4	119	66 (Med)	1981–2009	TLR-5	Fulfilled all items

REMARK, Reporting Recommendations for Tumor Marker Prognostic Studies; HPV, human papillomavirus; OPSCC, oropharyngeal squamous cell carcinoma; OSCC, oral squamous cell carcinoma; NPC, nasopharyngeal carcinoma; BOTSCC, base-of-tongue squamous cell carcinoma; TSCC, tongue squamous cell carcinoma; OTSCC, oral tongue squamous cell carcinoma; M, mean age; Med, median age.

**Table 2:** Summary of the staining and evaluation methods.

Study	Antibody Info	Dilution	Tissue	Scoring Grade	Cutoff Value
[10]	TLR-5: (mAb, Mo), Novus	1:100	TMA	Neg. 0, mild 1, moderate 2, strong 3	Values (1–3) are scored as positive
[12]	TLR-3: Abcam; clonality: ND	1:100	FFPE	Low, high	-
[17]	TLR-1: **(**Rb) Santa Cruz Bio. Inc. TLR-2: (pAb, Rb), Santa Cruz. TLR-4: (pAb, Rb) Santa Cruz. TLR-5: (mAb, Mo), Novus Biologicals. TLR-7: (pAb, Rb), Imgenex/Novus Biologicals. TLR-9: (pAb, Rb), Santa Cruz	TLR-1 1:100, TLR-2 1:200, TLR-4 1:300, TLR-5 1:100, TLR-7 1:300, TLR-9 1:100	FFPE	Neg., mild, moderate, or strong	It is required that >80% of tumour cells in the sample stained positively
[18]	TLR-5: **(**mAb, Mo), Imgenex TLR-7: **(**mAb, Rb), Imgenex	TLR-5 1:200 TLR-7 1:300	FFPE	Neg., weak, medium, strong	-
[11]	TLR-5: (mAb, Mo), Imgenex TLR-7: (mAb, Rb), Imgenex TLR-9: (pAb, Rb), Santa Cruz Bio. Inc.	TLR-5 1:200, TLR-7 1:300, TLR-9 1:100	TMA	0–3 (0 = none, 3 = strong)	Values 1–3 are scored as positive
[13]	TLR-4: **(**pAb, Rb) Boster Biological Tech Co.	1:150	FFPE	Low, high	IRS 3
[22]	TLR-1: **(**pAb), Abcam	1:100	FFPE	Percentage of stained cells 0–4	IRS: low 0–3, high 2–4. The (%) of stained cells was 0 for ≤ 25% staining; 1 for 25–50%; 2 for 50%; 3 for 51–75%; and 4 for more than 75% staining
[14]	TLR-7: Abcam; clonality: ND	-	FFPE	Grade 1–3	Grade 1 (1–3) Grade 2 (4–6) Grade 3 (7–9)
[19]	TLR-9: **(**mAb, Mo, IgG1), Imgenex	1:150	FFPE	Low, high	Histoscore: low 0–64, high 65–300
[20]	TLR-2: (pAb, Rb), Santa Cruz Bio. Inc. TLR-4: (pAb Rb), Santa Cruz Bio. Inc. TLR-5: (mAb), Imgenex. TLR-7: (pAb) Imgenex. TLR-9: (pAb), Santa Cruz Bio. Inc.	TLR-2 1:50, TLR-4 1:50, TLR-5 1:200, TLR-7 1:300, TLR-9 1:100	TMA	TLR-2, 4 and 7: 0–4 (0 = none, 4 = very high), TLR-5 and 9: 0–3 (0 = neg., 3 = strongly positive)	Low: none/mild High: mod./strong
[15]	TLR-4: Protein Tech & Affbiotect	1:150	FFPE	Over-expressed, under-expressed	50%
[16]	TLR-5: (mAb, Mo) (Imgenex)	1:100	FFPE	Low expression; high expression	7%
[21]	TLR-5: **(**mAb, Mo, IgG2a), Imgenex	1:150	FFPE	Weak and strong expression	Histoscore: weak 0–135; strong 136–300

FFPE, formalin-fixed paraffin-embedded; IRS, immunoreactive scoring; mAb, monoclonal antibody; Mo, mouse antihuman; Mod, moderate; ND, not disclosed; Neg, negative; pAb, polyclonal antibody; Rb, rabbit antihuman; TMA, tissue microarray.

**Table 3:** Summary of the prognostic data.

Study	TLR	Endpoint	Adjusted Analysis	Adjusted Factors	Results Interpretation
[10]	TLR-5	DSS	HR = 2.5, P = 0.129, 95% CI = 0.8–8.1	Gender, age, smoking, TN-class, stage, treatment, HPV status	High TLR-5 expression was an independent indicator of poor DSS in those with HPV-positive OPSCC. High TLR-5 was significantly associated with LN-status, tumour site and grade.
[12]	TLR-3	OS	-	-	TLR-3 expression was associated with poor prognosis and shorter OS. Higher TLR-3 was also associated with pathologic grade.
[17]	TLR-1 TLR-2 TLR-4 TLR-5 TLR-7 TLR-9	OS	TLR-7: HR = 0.37, P = 0.018, 95% CI = 0.16–0.84	Gender, age, ethnicity, smoking, TN-class, stage, histology, virus status, treatment, irradiation technique	Patients with positive TLR-7 tumour expression had better OS than those with no TLR-7 expression.
		DSS	TLR-7: HR = 0.39, P = 0.046, 95% CI = 0.15–0.98		
[18]	TLR-5 TLR-7	DSS	-	-	TLR-5 or TLR-7 did not have a statistically significant correlation with clinical outcome or survival.
		DFS	-		
[11]	TLR-5 TLR-7 TLR-9	DSS	TLR-7: HR = 3.2, P = 0.027, 95% CI = 1.1–9.0	Gender, smoking, TN-class, HPV status, treatment	High expression of TLR-5 and low expression of TLR-7 are correlated with poor DSS and RFS of HPV-positive patients.
		RFS	-		
[13]	TLR-4	OS	RR=2.334, P=0.006, 95% CI=1.277-4.267	TN-stage, adjuvant therapy, differentiation, invasion depth, cytoplasmic and nuclear NF-kBp65	High TLR-4 expression was an independent prognostic factor and significantly associated with lower DFS, DSS and OS. High TLR-4 expression was correlated with pTNM-stage, differentiation and invasion.
		DSS	RR=2.495, P=0.005, 95% CI=1.321-4.712		
		DFS	RR=2.888, P=0.001, 95% CI=1.532-5.443		
[22]	TLR-1	-	-	-	TLR-1 plays an inhibitory role in the development and progression of TSCC. High TLR-1 was correlated with TNM-staging.
[14]	TLR-7	OS	HR = 1.253, P = 0.547 (NS), 95% CI = 0.601–2.613	Gender, age, smoking, TNM-stage, differentiation, LNM, inflammation	High expression of TLR-7 in tumour cells correlated with shorter OS but not with DFS. On the contrary, high TLR-7 in stromal fibroblast-like cells was correlated with better survival time. High TLR-7 was also significantly associated with tumour differentiation.
		DFS	-		
[19]	TLR-9	CSS	HR = 1.810, P = 0.024, 95% CI = 1.053–3.112	Age, tumour stage, histologic grade	High TLR-9 expression was an independent predictor of poor CSS. TLR-9 correlates significantly with tumour grade.
[20]	TLR-2 TLR-4 TLR-5 TLR-7 TLR-6	OS	-	Pathologic T-stage, grade, presence of occult neck metastases, and invasion	Negative or mild TLR-5 expression was related to worse DSS.
		DSS	-		
		DFS	-		
[15]	TLR-4	POS	-	-	Patients with TLR-4 amplification had a shorter POS and high TLR-4 expression also correlates with T-stage, histological classification and metastasis.
[16]	TLR-5	DFS	-	-	TLR-5 expression was not associated with any clinicopathological characteristics or impact on survival.
[21]	TLR-5	DFS	HR = 3.587, 95% CI = 1.632–7.882	Gender, age, stage, histologic grade, adjuvant therapy	Strong TLR-5 expression was independent prognostic factor associated with reduced DFS and CSS.
		CSS	-		

CSS, cancer-specific survival; DFS, disease-free survival; DSS, disease-specific survival; HR, hazard ratio; LN, lymph node; LNM, lymph node metastasis; OS, overall survival; POS, post-operative survival; pTNM, pathologic tumour-node-metastasis; RFS, recurrence-free survival; RR, risk ratio; T., tumour; TLR, toll-like receptor; TN, tumour-node; TSCC, tongue squamous cell carcinoma.

**Table 4:** Results from the meta-analysis.

**Study**	**Number of Cases**	**Country**	**TLR Type**	**Endpoint**	**Hazard Ratio** **(95% CI)**	**Relative Weight, %**
**All Studies**						
[21]	119	Finland	TLR-5	DFS	3.59 (1.63–7.88)	2.00
[10]	143	Finland	TLR-5	DSS	2.50 (0.80–8.10)	3.09
[11]	331	Finland	TLR-7	DSS	3.20 (1.10–9.00)	16.33
[14]	166	China	TLR-7	OS	1.25 (0.60–2.61)	2.32
[17]	207	Finland	TLR-7	DSS	0.39 (0.15–0.98)	16.75
[17]	207	Finland	TLR-7	OS	0.37 (0.16–0.84)	30.51
[19]	197	Finland	TLR-9	CSS	1.81 (1.05–3.11)	29.00
Pooled overall estimate	0.96 (0.39–1.54)					100.00
Heterogeneity measures	I-squared = 61.8% (*p*-value = 0.0015), Tau-squared = 0.2533					
**Subgroup Analysis (TLR subtype)**						
**Study**	**Number of Cases**	**Country**	**TLR Type**	**Endpoint**	**Hazard Ratio** **(95% CI)**	**Relative Weight, %**
**TLR-5 Studies**						
[21]	119	Finland	TLR-5	DFS	3.59 (1.63–7.88)	57.70
[10]	143	Finland	TLR-5	DSS	2.50 (0.80–8.10)	42.30
Pooled overall estimate	3.13 (0.76–5.50)					100.00
Heterogeneity measures	I-squared = 0.0% (*p*-value = 0.657), Tau-squared = 0.000					
**TLR-7 Studies**						
[11]	331	Finland	TLR-7	DSS	3.20 (1.10–9.00)	0.91
[14]	166	China	TLR-7	OS	1.25 (0.60–2.61)	11.98
[17]	207	Finland	TLR-7	OS	0.37 (0.16–0.84)	47.27
[17]	207	Finland	TLR-7	DSS	0.39 (0.15–0.98)	39.84
Pooled overall estimate	0.51 (0.13–0.89)					100.00
Heterogeneity measures	I-squared = 34.6% (*p*-value = 0.205), Tau-squared = 0.0489					

DSS, disease-specific survival; OS, overall survival; TLR, toll-like receptor.

**Table 5:** Inclusion and exclusion criteria.

Inclusion Criteria	Exclusion Criteria
Original research articles	The retrieved records were case reports; reviews; letters; etc.
Histological tissue samples from human patients	Animal model studies and tests
Patients diagnosed with HNSCC	Articles not written in English language
Studies reported the association between TLR immunoexpression and the survival outcomes	Insufficient information of the correlation between clinical features and/or survival outcomes

HNSCC, head and neck squamous cell carcinoma; TLR, toll-like receptor.

## Supplementary Materials

Supplementary materials can be found at https://www.mdpi.com/1422-0067/21/19/7255/s1.

## Abbreviations

TLR	Toll-like receptor
HNSCC	Head and neck squamous cell carcinoma
OPSCC	Oropharyngeal squamous cell carcinoma
OSCC	Oral squamous cell carcinoma
NPC	Nasopharyngeal carcinoma
BOTSCC	Base-of-tongue squamous cell carcinoma
OTSCC	Oral tongue squamous cell carcinoma
