# Supplementary material for: The Prognostic Value of Toll-Like Receptors in Head and Neck Squamous Cell Carcinoma: A Systematic Review and Meta-Analysis
Source: Int J Mol Sci. 2020 Sep 30;21(19):7255. doi: 10.3390/ijms21197255 (PMC7582583; doi:10.3390/ijms21197255)
Supplement: Supplementary file 1 [file ijms-21-07255-s001.pdf]

Supplementary Materials

The Prognostic Value of Toll-Like Receptors in Head and Neck Squamous Cell Carcinoma: A Systematic Review and Meta-Analysis

Table S1. Evaluation criteria used to assess the reporting quality.

Checklist Items	Criteria †
1. Patient samples	Cohort (retrospective or prospective) study with a well-defined study population with information such as the number of the studied patients, source of sample, study period, follow-up time.
2. Clinical data of the cohort	The basic clinical data including gender, age, clinical stage of cancer and histopathological grade, was provided.
3. Immunohistochemistry	Well-described staining protocol or referred to original paper with information such as primary antibody name, dilution, company. The cut-off value of the area stained after which it is to be considered positive, was well described.
4. Prognostics	The endpoints of the survival analyses were defined (e.g., overall survival, disease-free survival).
5. Statistics	Estimated effects (HR, CI) were describing the relationship between the evaluated TLR(s) and the outcome was provided. Adequate statistical analysis (e.g., Cox regression modelling) was performed.
6. Classical prognostic factors	The prognostic value of the classical prognostic factors was reported. The relationship between the evaluated TLR(s) and classical prognostic factors were reported.

HR, hazard ratio; CI, confidence intervals. † Adapted from the Reporting Recommendations for Tumor Marker Prognostic Studies (REMARK) guidelines [1].

Table S2. Risk of bias appraisal and evaluation *.

Study	Q1	Q2	Q3	Q4	Q5	Q6	Q7	Q8	Q9	Q10	%	Risk of Bias
[10]	Y	Y	Y	Y	Y	Y	Y	N/A	N/A	Y	100	L
[12]	N	Y	Y	Y	U	Y	Y	N/A	N/A	N	62.5	M
[17]	Y	Y	Y	Y	Y	Y	Y	N/A	N/A	Y	100	L
[18]	Y	Y	Y	Y	U	Y	Y	N/A	N/A	N	75	L
[11]	Y	Y	Y	Y	Y	Y	Y	N/A	N/A	Y	100	L
[13]	Y	Y	Y	Y	U	Y	Y	N/A	N/A	N	75	L
[22]	Y	Y	Y	Y	U	Y	Y	N/A	N/A	N	75	L
[14]	Y	Y	Y	Y	U	N	Y	N/A	N/A	Y	75	L
[19]	N	Y	Y	Y	U	Y	Y	N/A	N/A	Y	75	L
[20]	Y	Y	Y	Y	Y	Y	Y	N/A	N/A	N	87.5	L
[15]	Y	Y	Y	Y	Y	Y	Y	N/A	N/A	N	87.5	L
[16]	Y	Y	Y	N	Y	Y	Y	N/A	N/A	Y	87.5	L
[21]	N	Y	Y	Y	U	Y	Y	N/A	N/A	N	62.5	M

* The evaluation was performed according to the (Meta-Analysis of Statistics Assessment and Review Instrument critical) appraisal tool as recently described [2]. The percentage indicates the “yes” score. Y = Yes, N = No, U = Unclear, NA = Not applicable (which was not considered on the percentage calculation).

References

1. McShane, L.M.; Altman, D.G.; Sauerbrei, W.; Taube, S.E.; Gion, M.; Clark, G.M. Statistics subcommittee of the nci-eortc working group on cancer diagnostics. Reporting recommendations for tumour MARKer prognostic studies (REMARK). *Br. J. Cancer* **2005**, *93*, 387–391.
2. Hujanen, R.; Almahmoudi, R.; Karinen, S.; Nwaru, B.I.; Salo, T.; Salem, A. Vasculogenic mimicry: A promising prognosticator in head and neck squamous cell carcinoma and esophageal cancer? A systematic review and meta-analysis. *Cells* **2020**, *9*, 507.
